# The Effectiveness of Progressive and Traditional Coaching Strategies to Improve Sprint and Jump Performance Across Varying Levels of Maturation within a General Youth Population

**DOI:** 10.3390/sports7080186

**Published:** 2019-07-30

**Authors:** Regan Standing, Peter Maulder

**Affiliations:** Centre for Sport Science and Human Performance, Waikato Institute of Technology, 3200 Hamilton, New Zealand

**Keywords:** PHV, sprint, jump, coaching, adolescent

## Abstract

Literature pertaining to youth development has identified the importance of understanding the physical, intellectual and emotional needs of adolescents prior to, during, and after their peak height velocity (PHV) period. The purpose of this study was to compare the use of a ‘traditional’ and ‘progressive’ coaching style to train a general male youth population to improve sprint and jump performances whilst assessing enjoyment to comment on long-term application. Maximal sprint times, sprint kinematics, unilateral jump distances and repetitive tuck jump scores were measured alongside anthropometric variables to characterise performance. The results revealed significant (*p* < 0.05) pre/post differences in anthropometric variables across all maturation groups, and each of the maturational levels displayed a tendency to favor a particular coaching or control condition. Pre-PHV groups responded most effectively to the progressive style of coaching, displaying improvements in horizontal jump performances, and −0.7% to −2.7% improvements in all sprint times, despite also showing the largest increase in tuck jump scores (25.8%). The circa-PHV group produced their greatest improvements in the traditional intervention, as displayed through significant improvements (*p* < 0.05) in 20-m sprint times and dominant-leg horizontal jump performance, whilst also revealing the greatest deterioration in tuck jump scores (14.2%). Post-PHV displayed the greatest improvements in the control setting, suggesting that the natural benefits gained through adolescent development were greater than the influence of the training interventions. In conclusion, the results suggest that matching coaching strategies and delivery techniques to the period of biological maturation may have implications for both performance and athlete safety.

## 1. Introduction

The use of long-term athlete development (LTAD) models has become widely discussed and implemented by coaches within sporting programmes working in youth settings [1,2,3]. These models aim to cater to the highly variable and non-linear nature of adolescent development by targeting age appropriate activity in an athlete-centered manner [4]. Youth coaches invested in these models promote discrete alterations in training focus throughout their sporting journey to allow individual growth and help create a positive relationship with exercise, ultimately aiming to preserve long-term participation [4]. The need for variety and individualisation within training regimes is critical due to the variable onset of peak height velocity (PHV) during the adolescent growth spurt. This gene-based, hormone-driven biological process dictates the rate and timing of physical and neurological maturation [5,6]. Due to the unpredictability in the length and intensity of this growth phase, it is common to see a large range of physical, psychological and emotional aptitudes within individuals of a similar chronological age [7]. Accompanying these changes is a rise in the risk of both structural and soft tissue injuries due to the increased rate of growth in bones and muscles [8,9]. Neurologically, this process includes the progressive myelination of axons, accompanied by synaptic and axonal pruning [10]. This development may be expressed through alterations in regular behavior, risk-taking, emotional responsiveness, as well as the individuals’ need for cognitive stimulation and sensation [11], and also through a phenomenon identified as ‘adolescent awkwardness’ which is used to describe the process of long bone growth prior to muscular growth, which can lead to a period of disruption in motor coordination [3]. The corresponding effects of these neurological and behavioral adaptations may have implications for learning needs, learning effectiveness and learning styles [12].

Previous research has investigated strategies such as athlete-centered learning [13], game sense [14], teaching games for understanding (TgfU) [15], and numerous types of coach-feedback strategies [16,17] aimed at optimising learning within a range of populations. Experimental studies investigating these topics have highlighted improvements in recognition performance, motor skills, emotional aptitude and decision making [18,19,20], which prompted further examination into their application in various contexts. Despite these investigations, there is limited research into the success of these strategies during arguably one of the most important developmental ages for youth: PHV. These pre-mentioned coaching strategies share key overlapping themes with slight variations in application, delivery and/or targeted outcomes. Key similarities between these strategies include the importance placed on the athletes need to interact, apply and discover learning for themselves, have fun, group interaction, problem solving, decision making and finally, learning through numerous interactions with technical, tactical or physical material in a range of contexts, which will collectively be referred to as ‘progressive’ coaching from here-on and so-forth. These methods are in contrast to a more traditional approach to coaching which typically encompasses technical drill-based methods, providing repetition and technical awareness for the individual prior to competing in the sport [21]. One particular study utilised a traditional style of coaching alongside a ‘strategy-orientated’ approach and identified that badminton serving skills improved most when taught utilising the traditional methods [22]. This approach provides an intimate context to teach, refine, and modify sport specific movements through repetition and exposure to the required technical skills [21,23,24], and may provide a more effective coaching style in some environments. 

Based on current literature surrounding the individual variation in physical, cognitive and emotional aptitudes within the adolescent population, a coach’s role is to ensure learning maximised through purposeful pursuits to stimulate the minds of youth via planned and strategic coaching methods. Previous successful applications of TGfU, game sense, athlete-centered coaching and also a traditional approach to coaching, suggest that their use throughout a range of movement contexts is warranted; however, they may be difficult to implement into some individual sports or training groups due to the lack of team and group interactions available, and the level of buy-in from coaches [21]. If learning and retention can be maximised within these cohorts of varying levels of biological maturation, then athlete independence, enjoyment, knowledge and physical longevity within sport can be improved; ultimately keeping adolescents interested in the sport for longer. 

The aim of this study was to build depth within the current pool of literature pertaining to youth and maturation. This study utilised two different coaching approaches (traditional and progressive) with the purpose of identifying the most effective strategy to improve sprint and jump performance within pre, circa and post-PHV maturation groups. These findings can be used to inform youth coaches and practitioners as to how they can interact with their cohorts to maximise learning and retention. Injury markers, movement kinematics and performance measures provide insight into alterations in movement that occur during the intervention, whilst enjoyment was measured to provide insight into athlete engagement. Due to the success of the TGfU and game sense approaches in different cohorts, it was hypothesised that a progressive coaching style would produce the greatest improvements in sprint and jump performance within the pre and post maturation groups when compared to the traditional coaching group, as well as display a decrease in injury markers. It was hypothesised that the circa maturation group would respond best to the traditional coaching methods, as the individual focus and direct feedback would limit the detrimental influence of adolescent awkwardness. Finally, it was hypothesised that enjoyment would remain consistent throughout both coaching strategies because of the short-term application of the intervention.

## 2. Materials and Methods

### 2.1. Study Design

This study utilised a semi-randomised test–retest design, which compared descriptive data from three distinct maturation groups (pre, circa, and post-PHV), under three separate conditions (traditional coaching, progressive coaching, and control), within the targeted male youth population of a single high school. Those individuals within the control and training groups were pre-determined (non-randomised) due to schooling physical education class allocation, however the traditional and progressive groups were allocated by using a random number generator which selected even distributions of the three maturation groups. These maturation groups were allocated after the initial pre-testing with the use of a sex-specific PHV calculation [7] which utilises height, seated height and limb length, to measure maturation offset (pre < −0.50, circa −0.49 to +0.49, post > +0.5) [25]. Despite this equation having a reported variance of ±0.592 years [26], the allocations were made in accordance with similar studies [26,27] to allow better distribution across maturation groups within this population.

### 2.2. Participants

A total of 111 youth males (aged 13.2–15.7 years; maturity offset −1.0 to 2.6 years) from a single high school volunteered for this project. A completed health questionnaire with no contraindications and guardian consent were required to partake in this study. There were no fitness or sporting requirements for the participants, as a representation of general youth ability was sought. Due to the use of a single high school, there was a mix of athletic and non-athletic individuals within the tested population. Inclusion criteria for data analysis required pre and post testing completion, in addition to an 80% completion of training sessions for the training groups. These criteria led to a 25.2% dropout from the initial 111 volunteers (traditional = 9.9%, progressive = 7.2%, control 8.1%). Full data sets were recorded for a total of 83 participants (traditional n = 28, progressive n = 30, control n = 25). Table 1 displays the group characteristics per training and maturation group. Ethical approval was granted from the Waikato Institute of Technology ethics committee for all procedures from the institutes’ ethics committee.

### 2.3. Experimental Procedures

Both the training and control groups were required to attend a pre- and post-testing session which lasted approximately 50 min each and were separated by a six-week period. Additionally, training groups participated in five training sessions lasting between 40 and 50 min each, depending on school timetabled class durations. All sessions were performed bare feet on a wooden gymnasium floor in self-selected active wear. A standardized warm up was led prior to each session, which lasted approximately 12 min and consisted of dynamic, progressive exercises targeting the whole body initially, then the lower limb specifically. Familiarization occurred prior to the commencement of each pre and post-test via verbal instruction and a visual demonstration. Each participant was provided the opportunity to practice each movement prior to the recorded trials.

The five training sessions utilised with both the traditional and progressive training groups aimed to improve sprint technique via several mechanical factors including body positioning, lower limb mechanics, upper limb mechanics, and ground contact characteristics [28,29,30,31]. The traditional and progressive coaching strategies were characterised by several key strategical differences (Table 2) with technical aspects derived from previous literature [28,32,33].

Each session, the two coaches would change the group they delivered to in order to ensure that there was no bias towards personal delivery characteristics that may influence the PACES survey and enjoyment outcomes. Both coaches had been coaching youth sport for a minimum of eight years and were current coaches in the industry. Each coach consciously focused on a fun and engaging delivery style which included variable tone and pitch in voice, open body-language, and a high level of energy, irrespective of whether they were with the traditional or progressive group, to ensure differences were only evident in the pre-determined coaching strategies (Table 2).

### 2.4. Data Collection

#### 2.4.1. Anthropometrics

Height, seated height and body mass were measured during pre-testing to provide information for the PHV calculation [7]. Standing height was measured via a free-standing stadiometer, with the participants feet shoulder width apart and the chin and line of sight parallel to the floor. The headpiece was lowered firmly on the center of the participants’ heads whilst they were standing with an erect posture. Seated height was measured whilst they sat on a 30 cm anthropometric box placed against a wall with a tape measure aligned vertically from the center of the box. Participants had their legs together and hands rested on their knees. The lower back was firmly against the wall at the rear of the box and the chin and eye line were parallel to the floor. The headpiece was lowered firmly on to the participants head, ensuring that a right angle was kept with the wall. Both standing and seated heights were measured to the nearest mm. Body mass was taken on a set of electronic scales which were zeroed prior to each participant’s measurement.

#### 2.4.2. Sprint Performance

Participants performed three maximal effort 20-m sprints (2 min rest between each trial), utilising a standing split stance with their preferred foot placed on the starting line 0.5 m back from the first timing light [34]. A dual-beam-modulated SWIFT timing light system (Wacol, Brisbane, Australia), captured performance times using four sets of lights placed at the zero, 5 m, 10 m and 20 m marks, at a height of 0.85 m (to top of tripod), with the lane width was approximately 3 m. The initial timing light gate (0 m) was set lower (65 cm to the top of tripod) than the other gates to account for the likely hunched starting positions of the participants. Each trial began with a forward movement of the torso, as opposed to a rocking motion where the momentum could be generated prior to first foot movement. Once instructed to step up to the line, the participants were free to commence the trial in their own time to remove any variability in the reaction times.

#### 2.4.3. Sprint Kinematics

Two high-speed cameras (Casio Exilim, ex-zr200, Las Vegas, NV, USA) capturing at 240 fps on fixed tripods (set at 0.8 m to base of tripod) were placed to capture a sagittal view perpendicular to the line of sprint. Camera one was set at a 2.5 m distance from the start line and 6 m perpendicular to the center of the runway, which allowed the capturing of the first 5 m of each sprint. Camera two was set at the 15 m mark, 9 m perpendicular to the runway with a field of view at approximately 12.5–17.5 m of the line of the sprint. Calibration markers (1.5 m in length) were placed central to both cameras to replicate similar distances to those observed in comparable populations within the relevant literature [35] and to minimize parallax error. Data analysis of the sprint kinematics required the use of Silicon-coach pro 7 (Dunedin, New Zealand) to measure the following variables, with metrics derived from the recommendations of [36]:

- Step length (m)—Horizontal distance between the point of touchdown of one foot (furthest point) and the touchdown of the following foot.
- Step rate (Hz)—The number of steps per second, calculated via the following equation: 1/(stance + flight time).
- Stance time (s)—Duration of the time taken from the last frame before contact with the ground to the last frame with contact.
- Flight time (s)—Duration of the time taken from the last frame displaying contact with the ground to the frame prior to ground contact.

#### 2.4.4. Unilateral Horizontal Jumps

Maximal unilateral horizontal jump performance was obtained via three jumps for distance from each leg (take-off one leg and land with two), with an approximately 2-min rest between trials [37] (alternating legs each trial). Measurements were taken from the rear-most heel on a successful landing [38]. An unsuccessful landing consisted of an individual falling backwards, stepping backwards, or putting their hands down behind the rear-most heel (these trials were repeated). Hands were free to move throughout the movement and no coaching or technical cues were given. 

#### 2.4.5. Tuck Jump Assessment

A single 10 s bilateral tuck jump (TJ) assessment was performed and qualitatively marked against a modified rubric (Appendix A) [39]. Intra-rater reliability statistics (ICC) for the modified TJ assessment was calculated at 0.971 (substantial) and a 93% PEA, with Kappa scores ranging from 0.615 to 1.00 (*p* < 0.05) for each of the 10 individual variables within the rubric. On the gym surface where the test was to be performed, tape was used to create a box with edges 41 cm in length and 35 cm wide, which the participants were instructed to remain on if possible [39]. This assessment required the participants to perform continuous tuck jumps for a period of 10 s within the specified area (if possible). Instructional cues consisted of the following: “bring knees to chest”, “continuous jumps for 10 s”, and “jump as high as you feel comfortable”. Two high-speed cameras (Casio Exilim, ex-zr200, Las Vegas, NV, USA) capturing at 120 fps on fixed tripods (set at 0.8 m to base of tripod) provided frontal and sagittal views of the participants during their tuck jump assessments. Scores were allocated via post-session video analysis and compared against a severity based kinematic marking criteria (Appendix A). It is important to note that the risk factors for injury are multifactorial, and are therefore likely to differ based on different types of injuries and sports. Although the TJ assessment provides insight into several injury markers (trunk dominance, quadriceps dominance, neuromuscular fatigue, leg dominance, ligament dominance, feedforward mechanisms deficit) it is unlikely that this one-off assessment accurately predicted injury risk; rather, it can aid in identifying potential areas to improve to decrease this risk.

#### 2.4.6. Paces Survey

Enjoyment levels for both training groups were sought through a PACES questionnaire [40] derived from [41], which was administered approximately five minutes after the completion of the final session. Instructions were to fill out the survey as honestly as possible and to take the time to read and think about each question carefully.

### 2.5. Statistical Analysis

A post-only spreadsheet from Hopkins [42] was utilised to analyse pre/post changes within maturation levels across training groups for all performance measures and kinematic variables. Specifically, differences were expressed as percentage via analysis of log-transformed values using natural logarithms. Logarithmic transformation allows for the uniformity of error, thus, this strategy was used to reduce bias arising from non-uniformity of error (differences) of the variables of interest. Differences between log-transformed measures are expressed as percentage differences, with effect sizes, 90% confidence limits, *p* values and qualitative inferences used to supplement these changes. A difference was deemed *unclear* if confidence limits of the effect statistic overlapped zero. If a result was deemed as *clear*, effect sizes were awarded per the descriptors of Hopkins [43]; 0–0.2 *trivial*; 0.2–0.6 *small*; 0.6–1.2 *moderate*; 1.2–2.0 *large*; 2.0–4.0 *very large*. Statistical significance was awarded for variables with a *clear* effect size and *p* < 0.05.

The mean of the two best sprint and horizontal jump trials was utilised for each participant and used as comparative scores as per the recommendations of Maulder, Bradshaw and Keogh [44]. Further statistical analyses compared change scores for the 5 m, 10 m, and 20 m sprints, as well as the HJD, HJND, TJ score, and kinematic variables across maturation levels between control, traditional and progressive training groups. A spreadsheet for the analysis of pre-post parallel groups’ trials [45] was utilised to derive net percentage changes, *p* values, 90% confidence limits, and effect sizes, whilst qualitative descriptors were used to describe effect sizes [46]. A difference was deemed unclear if confidence limits of the effect statistic overlapped zero. If a result was deemed as *clear*, effect sizes were awarded per the descriptors of Hopkins [43]; 0–0.2 *trivial*; 0.2–0.6 *small*; 0.6–1.2 *moderate*; 1.2–2.0 *large*; 2.0–4.0 *very large*. Statistical significance was awarded for variables with a *clear* effect size (ES) and *p* < 0.05.

The PACES enjoyment survey was analysed via a spreadsheet comparing group means [47]. This provided mean and standard deviations for both training groups accompanied by *p* values and effect sizes to interpret the magnitude of difference [43].

## 3. Results

### 3.1. Anthropometrics and Performance Measures

Pre- and post-test means and SD for sprint and jump metrics can be found in Table 3 and Table 4. Log-transformed within-group differences and between-group differences can be observed in Table 5, Table 6, Table 7 and Table 8.

Pre-testing data identified that there were no significant differences (*p* > 0.05) between training-groups of the same maturation level prior to intervention. It was observed that height, body mass and seated height increased significantly for all training groups (*p* < 0.05) over the five-week intervention without maturational grouping. The exception to this was the control-group seated height which had a non-significant trivial-small increase (0.4% ± 90%CL = 0.9%; *p* = 0.479). Maturational grouping displayed that the pre PHV and circa-PHV groups significantly increased height, body mass and seated height (*p* < 0.05) during the intervention period, with the post-PHV group showing significant differences in height (0.9% ± 0.7%, *p* = 0.035) and body mass (2.2% ± 0.8%, *p* < 0.001) only. 

When comparing pre/post change scores, it was revealed that small-large significant differences in TJ scores between the control and progressive-groups (*p* = 0.018) were evident. No significant differences were observed for any anthropometric, sprint or horizontal jump measures when maturation was utilised as a covariate and compared across training-groups (see Table 7 and Table 8). Despite being non-significant, clear outcomes were identified for many performance-based metrics.

When comparing strictly pre-PHV means between training-groups, clear outcomes were identified for the progressive-group who displayed the largest change in mean 5 m (−2.1% ± 2.9%, *p* = 0.080), 10 m (−1.1% ± 2.7%, *p* = 0.395), and 20 m (−2.7% ± 3.2%, *p* = 0.136) sprint times, with effect sizes ranging from trivial to moderate (see Table 5). Group sprint means (5 m, 10 m, and 20 m) for both the traditional and control pre-PHV groups were up to 4.4% slower when compared to pre-assessment times (see Table 5). This trend continued within the jump data, with the progressive-group pre-PHV eliciting trivial to large improvements in HJD (10.8% ± 10.7, *p* = 0.098) and HJND (11.0% ± 6.2%, *p* = 0.027) performances (see Table 4). Despite the traditional and control-groups also eliciting positive jump performances (4.3% to 7.6%), effect sizes were unclear—moderate and statistically non-significant (*p* > 0.05). Contrasting to these results, pre-PHV tuck jump scores showed the largest deterioration within the progressive-group (25.8% ± 22%, *p* = 0.073), with the traditional and control-groups improving their scores by 15.6% ± 84.9% (*p* = 0.547), and 11% ± 49.7% (*p* = 0.506), respectively (see Table 4 and Table 6).

When comparing circa-PHV groups, decreased sprint times were observed in each of the 5 m, 10 m, and 20 m distances across all training-groups, with mean improvements of −0.1% to −3.1% (see Table 3 and Table 5). The circa-PHV progressive (−1.6% ± 1.2%, *p* = 0.043) and control (−2.2% ± 1.7%, *p* = 0.036) 20 m sprint times were the only statistically significant improvements in sprint times, both with trivial to small effect sizes. Although non-significant, the traditional-group elicited the greatest improvements in circa-PHV HJD (10.1% ± 4.9%, *p* = 0.008), and HJND (9.9% ± 8.2%, *p* = 0.060) scores, but as seen in the pre-PHV groups, the training-group who witnessed the greatest gains in horizontal jump distance also displayed the greatest deterioration in TJ score (14.2% ± 29.1%, *p* = 0.350), in contrast to the control group, who improved by 8.9% ± 13.4 (*p* = 0.213) (see Table 6). 

When comparing post-PHV change scores, unclear results were identified for all training-groups for 5 m sprint times, with pre/post change scores ranging from −0.9% to 0.7%. Post-PHV 10 m sprint times displayed trivial to moderate improvements for the traditional (−2.1% ± 2.7, *p* = 0.177) and control (−0.9% ± 1.7%, *p* = 0.321) groups, with the progressive-group slowing by 0.6% ± 1.6% (*p* = 0.538). Significant improvements were identified in control (*p* = 0.028) and traditional (*p* = 0.030) 20 m sprint times, with the progressive-group improving by a non-significant −0.3% ± 1.9% (*p* = 0.748). All HJD and HJND performances improved significantly (*p* < 0.05) between 3.8% and 9.3% at the post-PHV level across all training groups, with all TJ scores increasing between 1.9% and 12.9% (see Table 4 and Table 6).

When removing maturation as the covariate and observing training groups in their entirety, there were significant changes for several sprint and jump performance measures. Trivial to small improvements were seen in the control (−1.8% ± 1.1%, *p* = 0.008), and traditional (−1.8% ± 1.1%, *p* = 0.008) 20 m sprint times, as well as small to moderate improvements in HJD and HJND performances for all training groups, irrespective of maturational grouping (*p* < 0.05) (see Table 4 and Table 6). 

### 3.2. Kinematic Measures

Whilst incorporating maturation and comparing training-group kinematic characteristics, there were several significant changes within the circa and post-groups, with no significant (*p* > 0.05) differences between training-group kinematic variables at the pre-PHV level (see Appendix B). 

The circa-PHV progressive-group measures displayed significantly larger 15 m flight times (14.3% ± 8.9%, *p* = 0.015), and significantly lower step frequencies at the second-step (9.5% ± 8.0%, *p* = 0.036) and 15 m-step (9.8% ± 7.8, *p* = 0.028) when compared to the circa-PHV control-group (see Appendix B). During step-two, the circa-PHV control group displayed an increase in step-length (7.7% ± 6.1%, *p* = 0.038) and shorter flight time during step-one (−34.3% ± 22.8%, *p* = 0.026) when compared to the traditional-group change scores. Significant (*p* < 0.05) small-large effect sizes were identified between the circa-PHV traditional and progressive contact times at step-two (9.3% ± 6.9%) and 15 m (7.7% ± 5.3%), as well as step-frequency during step-two (8.8% ± 6.5%) (see Appendix B). 

Comparing between groups at the post-PHV level, control-groups displayed trivial-moderate longer step length during step-two (*p* = 0.039) and three (*p* = 0.041) when compared to both progressive and traditional-groups, respectively (see Appendix B). The post-PHV control-group also displayed a shorter contact time during step-one (−6.9% ± 5.5%, *p* = 0.031) when compared to the progressive-group, and a lower step frequency during step-one (13.7% ± 8.1%, *p* = 0.010) when compared to the traditional group. The post-PHV traditional-group displayed a trivial to moderate difference in step-one step-length in comparison to the progressive-group (*p* = 0.045).

When maturation was removed as a covariate and training groups were analysed in their entirety, significant differences in step frequency were observed between control and traditional-groups during step-one (*p* = 0.032). It was also determined that the traditional-group had a significantly faster contact time at the 15-m mark (−5.4% ± 3.7%, *p* = 0.018) than the progressive group.

Despite being non-significantly different to improvements witnessed in other training groups, pre/post comparisons revealed that the control-group had a significant increase in step 2 step-length (4.7% ± 2.1%, *p* = 0.001), accompanied by a trivial to moderate increase in contact time during step-one (0.217–0.224 s, *p* = 0.025). 

Significant decreases were observed in traditional-group contact time (*p* = 0.001, ES = small-moderate) and flight time (*p* = 0.019, ES = trivial-small) at the 15-m recording, with mean changes ranging from −5.8% to −7% for both observed metrics.

The progressive group significantly increased mean 15-m flight time (0.091–0.098 s, *p* = 0.029, ES = trivial-moderate) and 15-m step length (1.70–1.75 m, *p* = 0.023, ES = trivial-small) over the course of the intervention.

The PACES enjoyment survey revealed no significant differences within maturation and coaching groups (*p* > 0.05), with mean scores ranging from 49.7 to 61.4. The circa-PHV group displayed the only clear difference between traditional and progressive coaching methods, with the progressive being identified as more enjoyable with a trivial-large effect size (*p* = 0.090).

## 4. Discussion

The purpose of this study was to compare the effects of a progressive and traditional coaching style on sprint and jump performance within varying levels of maturation. Previous literature informed the hypothesis that the progressive group would elicit the greatest sprint and jump improvements for the pre and post-PHV groups in conjunction with a decrease in injury markers. Based on the phenomenon termed ‘adolescent awkwardness’, the circa-PHV group was hypothesised to respond best to the traditional style of coaching, whilst enjoyment would be consistent between traditional and progressive groups regardless of maturation. As hypothesised, the results of this study revealed that although non-significant (*p* > 0.05), different coaching modalities may elicit superior improvements in sprint and jump performances if delivered to those of the appropriate physical and neurological maturation; however, increases in performances requiring high force generation may correspond with a heightened risk of injury. 

### 4.1. The Effects of Progressive and Traditional Coaching Strategies on Pre-PHV Groups

The progressive coaching style promoted the greatest improvements in 5 m (−2.1% ± 2.9%), 10 m (−1.1% ± 2.7%), and 20 m (−2.7% ± 3.2%) sprint times, and both horizontal jump performances (HJD (10.8% ± 10.7), HJND (11.0% ± 6.2%)), when compared to the traditional and control groups (see Table 5 and Table 6). This indicates that this method of coaching may in fact benefit the pre-PHV maturation-group more than the other styles if performance is the desired outcome. This finding supports both the hypothesis of the current study and the relevant literature surrounding the underlying methods incorporated into the progressive coaching style [16,20,21,48]. A meta-analysis completed by Moran, Sandercock, Rumpf and Parry [49], investigated sprint enhancement with respect to maturation and describes how improvements in pre-PHV sprint performances are typically restricted due to the limitations surrounding muscular strength, neuromuscular control and anthropometric factors. The current study produced dissimilar findings to these, and although results are non-significant, they suggest that appropriate coaching strategies may produce viable sprint training opportunities within the pre-PHV population. The disparities between the meta-analysis performed by Moran, Sandercock, Rumpf and Parry [49] and the current study lie in the style of intervention, and the population tested. Inclusion for the study by Moran et al., [49] required sprinting-based movements with a specific recovery period and utilised participants who were engaged in organised sport. In contrast, the current study used sub-maximal fundamental sprint mechanics as the training intervention aimed at altering technique within a general population of individuals. These factors may be critical in identifying when and how to target sprint training within the pre-PHV population. 

When investigating the mechanisms behind the sprint improvements, previous research has identified that improved sprint times involve increases in step length and/or step frequency without negatively effecting the other [50,51]. Kinematic analysis of the pre-PHV progressive group means supported these statements as increases (*p* > 0.05) in step length were evident, with little variation in step frequency when compared to pre-test measures (see Appendix B). Previous literature has linked a longer step length to increases in standing height and limb length [1,26], both of which increased significantly (*p* < 0.05) within all the pre-PHV groups over the period of the intervention. These anthropometric variations begin to provide a plausible mechanism for the altered kinematics; however, it is important to note the traditional and control groups also exhibited these anthropometric trends, but unlike the progressive group, these did not transpire to improved step length and/or frequency. This conclusion acknowledges the plausibility of the successful application of the progressive coaching sessions, which focused on key sprint mechanics and movement efficiency, ultimately refining and synchronising movement patterns more than the traditional or control groups [28,33,49]. The ability to coordinate the sequencing of multiple limb segments, synchronise motor unit recruitment, and increase the number of motor units utilised has been shown to produce greater muscular force output [30,52]. These physiological and neural adaptations can be gained through muscular overload and high velocity muscular activation [53,54], the latter being a specific element included in the training programmes utilised in this study. Supporting this hypothesis, the HJD and HJND displayed significant increases in jump distance, which illustrates a likely increase in lower limb power [55,56,57], which has been shown to be an important factor in improving sprint performance [58,59]. It is unwise to state that improved lower limb power via neural activation, or neuromuscular adaptation, is a leading cause of performance and kinematic improvements in the current study due to the lack of specific measurements of these variables; however, due to the short duration and power-based tests performed, it is a conclusion worth considering. 

This notion of increased muscular output is further supported by the findings in the pre-PHV tuck jump scores, which showed the largest decrement in the progressive group, suggesting that they had an increased risk of injury post-intervention. The need to safely control and decelerate limbs via eccentric contractions is vital to injury management, and can be exasperated during periods of increased force production [37,60]. This process requires an element of technical control and muscular strength, neither of which were targeted during the coaching sessions of this intervention. These findings suggest that the improvements in sprint and jump performances witnessed within the pre-PHV group were accompanied by a decreased ability to safely control the underlying mechanisms responsible for these improvements. This finding is critical in the long-term safety of athletes, as previous research has already identified a higher injury rate for individuals around the period of PHV [8,61,62,63]. Future interventions pursuing sprint and jump improvements should consider eccentric, plyometric and/or other strengthening interventions to supplement their sprint and jumps training to not only increase the performance response, but also to provide the technical and physical proficiency required to safely accommodate the physiological changes that occur during this process [55,64,65].

### 4.2. The Effects of Progressive and Traditional Coaching Strategies on Circa-PHV Groups

Based on the data collected, it is ill-advised to state that the circa-PHV group responded more effectively to any one of the training methods utilised within this study, therefore proving the initial hypothesis to be incorrect. Despite the lack of significant findings, the circa-PHV traditional group displayed the greatest improvements in 5 m (−1.1% ± 3.3%) and 20 m (−3.1% ± 3.3%) sprint times, as well as both the horizontal jump distances. This trend may begin to reveal an underlying need to adjust coaching strategies between levels of maturation. The traditional approach incorporated direct, individual feedback, as opposed to the previously successful questioning and problem-solving methods used within the progressive style of coaching [16,20,21,48]. The poorly understood, yet frequently acknowledged phenomenon termed adolescent awkwardness [1,66,67], may be influential in explaining why the traditional training was successful within the circa-PHV population. Adolescent awkwardness occurs during the adolescent growth phase and is characterised by rapid long-bone growth prior to muscular development, which may correspond with a period of disruption in motor coordination [3,67]. Clear, direct, and individual instructions, such as those utilised in the traditional coaching method, may help to produce a more effective movement output [68,69], or minimise the supposed disconnect between the brain and body during the adolescent growth spurt more than the strategies observed within the progressive coaching style. 

When analysing sprint metrics, all circa-groups improved in each of the 5 m, 10 m and 20 m sprint times, albeit insignificantly for the majority (see Table 7). Kinematic variables associated with these sprint performances show the traditional and control groups displaying non-significant (*p* > 0.05) increases in most step length and step frequency measures, which supports the findings of past sprint literature [36,50,51]. This tendency proved inconsistent within the progressive group who increased step length in all measured ground contacts, but also saw a decrease in step frequency throughout. These discoveries suggest that this decrease in step frequency was not enough to inversely affect the performance gains achieved through the increased step length, or inform that there were other factors at play outside of this studie’s measured variables [51,70]. As discussed, the kinematic variations across groups are likely influenced by the significant increases (*p* < 0.05) in standing height, body mass and seated height observed for all the circa-PHV groups as a natural response of maturation [1,26]. It is important to acknowledge that there likely are factors external to the study design that were influential to sprint results within this population. It was hypothesised that varying levels of cognitive focus, fatigue and motivation [71,72], movement experience gained through incidental exercise or regular physical education classes or neuromuscular maturation may have influenced the overall findings [73,74]. 

As observed within the pre-PHV findings, the training approach that generated the greatest sprint and jump improvements within the circa-PHV population also produced the greatest increase in injury markers during the tuck jump assessment. This trend has been hypothesised to be attributed to increases in concentric power, segment sequencing and/or the inability to accommodate the increases in these physiological alterations. To counter these initial statements, the control group improved their tuck jump score by 8.9%, which implies that they were at a decreased risk of injury than their pre-test; however, they also improved each of their sprint times, which suggests that the mechanism behind these variations is still unclear and requires further investigation. It is recommended that this test be utilised with caution until the underlying causes of these changes are identified within this population [75].

### 4.3. The Effects of Progressive and Traditional Coaching Strategies on Post-PHV Groups

As discussed previously, the lack of significant group differences within maturation suggests minimal differences between coaching strategies and sprint performances. Despite this, the control post-PHV group elicited the greatest improvements in 5 m and 20 m sprint times, as well as both horizontal jump distances and tuck jump scores (see Table 5 and Table 6). These results counter the initial hypothesis of this paper and suggest that neither of the training groups were able to generate performance benefits greater than those achieved through natural maturation, rendering the training intervention ineffective within this population. Biological maturation within the post-PHV includes hormonal, physical, neurological and physiological adaptations that result in a greater muscle mass, increased long bone length, and neural enhancement, which lead to natural improvements in some motor tasks [1,26] and in sprint performance [49]. These statements are supported by control groups producing comparable improvements in sprint performances to those observed in both training groups, accompanied by significant increases (*p* < 0.05) in standing height and body mass. Despite these increases, step length and step frequency displayed irregular but similar changes through all training and control groups, therefore suggesting that their influence on sprint performance was limited within this cohort [36,50,51]. Probable justifications for these increases in sprint times and horizontal jump performances include refined neuromuscular coordination and increases in muscular output and/or greater mechanical efficiency [30,52], although without direct measures of these variables, it is difficult to conclude. 

Based on the findings of the current study, technical training utilising traditional or progressive coaching methods is not sufficient to elicit responses greater than those achieved through natural maturation, and therefore trainers and coaches working with individuals of post-PHV maturation should employ appropriate physical interventions alongside technical training of various nature to maximise motor improvements. As per the recommendations of [2] and [73] interventions targeting plyometric and resistance, training exercises may elicit responses within the post-PHV maturation group than movement-based coaching alone. It is important to note that coaches working with adolescent athletes need to acknowledge the impact of physical and neurological maturation when comparing performances or pre/post testing in sporting contexts, especially if it is to provide a measure of training effectiveness for new athletes, as these improvements may in fact be due to natural maturation and not be a consequence of training strategies. 

### 4.4. Collective Group Findings

When comparing training groups within maturation levels, there were no significant differences (*p* < 0.05) in pre/post change scores between training groups and control groups. It is hypothesised that these findings may be due to the lack of statistical power from low participant numbers within the pre-PHV group and the overall variance witnessed due to the general population utilised within this study. 

As hypothesised, enjoyment played a limited role when it came to training group selection, as results proved that there were no significant differences (*p* > 0.05) within maturation levels. Mean scores ranged from 49.7 to 61.4 points (out of a maximum of 80), suggesting that there was an adequate level of enjoyment through each training modality; therefore, over a five-week period, either strategy is appropriate from an enjoyment perspective and performance gains will provide justification for using one approach over the other. 

### 4.5. Limitations and Future Recommendations

A primary limitation of this study was the low participant numbers within the pre-PHV groups. This was due to the ages of the high school students utilised and the need to break a small pre-PHV cohort into three different experimental groups. Despite this, training groups within pre-PHV maturation were of a similar size, allowing a more consistent statistical approach to be applied. Future research should utilise a slightly younger cohort to provide greater pre-PHV numbers and improve the statistical strength of the analysis. Secondly, the PHV equation used to separate maturation groups, as presented by Mirwald, Baxter-Jones, Bailey and Beunan [7], has had a reported variance of ±0.592 years [27]. These findings suggest that individuals who are within this acknowledged range could be wrongfully grouped, ultimately decreasing the clarity of the results and likely affecting the significance of the findings. Future recommendations regarding this concept include utilising a greater diversity of ages to provide a more distinct maturational difference between groups. In addition, training studies aiming to improve sprint performance through muscular and neural enhancements should incorporate protective elements to allow the safe dissipation of forces and eccentric control required to accommodate any power developments. Future recommendations also include the quantification of extra-curricular exercise, physical education classes and sports trainings in order to help clarify the differences between training adaptations, and those gained as a natural consequence of biological maturation. Final recommendations of this study include the need for strengthening exercises to help decrease the risk of injury encountered within movements requiring repetitive high-force outputs. This could be pursued through resistance training or plyometric interventions, or possibly through movement-based coaching strategies. With the lack of significant differences between groups, accompanied with sprint and jump performance, improvements throughout maturation levels and training groups, it is recommended that a variety of coaching methods be used to target individual learning styles if a movement-based sprint intervention is being implemented.

## 5. Conclusions

A summary of the findings from the current study has revealed a variety of aspects worthy of consideration when implementing intervention and coaching strategies across various levels of maturation. The use of a progressive coaching style incorporating elements of problem solving, competition, group interaction and guided feedback has shown to be more effective for individuals within the pre-PHV growth-phase. This was inconsistent between maturation levels, as the circa-PHV responded more effectively to the traditional coaching style that incorporated direct individual feedback focusing on repetition and self-improvement, likely influenced by the impact of adolescent awkwardness. Finally, the post-PHV group showed a less effective response to the training groups than they did to the natural benefits gained throughout natural biological maturation in the control group. These findings suggest that varying levels of biological maturation may require the use of unique coaching strategies in order to prompt the most effective outcomes from training programmes being implemented. It is also imperative to re-iterate that natural improvements in movement-based activities are likely during biological maturation, and coaches working with these athletes need to acknowledge these when quantifying the effectiveness of any training interventions.

## Figures and Tables

**Table 1:** Descriptive anthropometric statistics for training and maturation groups (Mean ± standard deviation (SD)).

Maturation Group	Training Group	N	Age (year)	Height (cm)	Body Mass (kg)	Maturity Offset (year)
Pre-PHV	CT	3	13.5 ± 0.2	155.7 ± 1.5	43.1 ± 2.1	−0.8 ± 0.2
	Trad	4	13.9 ± 0.7	154.7 ± 2.9	45.4 ± 3.1	−0.7 ± 0.1
	Prog	4	13.5 ± 0.7	156.8 ± 5.3	49.4 ± 4.5	−0.7 ± 0.1
Circa-PHV	CT	14	14.1 ± 0.7	163.4 ± 5.3	52.2 ± 8.0	0.0 ± 0.3
	Trad	7	14.1 ± 0.5	162.7 ± 6.3	53.4 ± 10.3	0.1 ± 0.3
	Prog	10	14.2 ± 0.5	165.1 ± 4.4	54.4 ± 7.7	0.0 ± 0.2
Post-PHV	CT	8	14.7 ± 0.7	173.3 ± 7.2	59.2 ± 6.7	1.3 ± 0.4
	Trad	17	14.7 ± 0.5	173.3 ± 6.1	62.9 ± 10.2	1.2 ± 0.6
	Prog	16	14.8 ± 0.4	172.7 ± 5.7	66.0 ± 8.2	1.2 ± 0.5

Note: CT = Control group; Trad = Traditional group; Prog = Progressive group.

**Table 2:** Strategical differences of the traditional and progressive coaching styles.

Traditional	Progressive
Coach led	Coach and athlete led
Provided information to athlete	Guided athletes to discover learning
Individual feedback given to athletes	Feedback provided through individual questioning and group discussion
Activities and drills performed individually	Group and pair activities used
Focus on individual skill improvement	Focus on group culture and interaction
Repetition and technical focus	Problem solving required
No group-based competition	Competition within group

**Table 3:** Pre- and post-sprint mean ± SD for training and maturation groups.

Metric	Maturation Group	Test	Control Mean ± SD	Traditional Mean ± SD	Progressive Mean ± SD
5 m (s)	All	Pre	1.16 ± 0.08	1.15 ± 0.07	1.16 ± 0.08
		Post	1.16 ± 0.08	1.15 ± 0.07	1.16 ± 0.07
	Pre-PHV	Pre	1.15 ± 0.04	1.17 ± 0.05	1.21 ± 0.07
		Post	1.18 ± 0.05	1.22 ± 0.06	1.18 ± 0.08
	Circa-PHV	Pre	1.18 ± 0.09	1.19 ± 0.10	1.18 ± 0.05
		Post	1.18 ± 0.09	1.18 ± 0.06	1.18 ± 0.05
	Post-PHV	Pre	1.13 ± 0.04	1.12 ± 0.05	1.14 ± 0.10
		Post	1.12 ± 0.07	1.12 ± 0.06	1.15 ± 0.09
10 m (s)	All	Pre	2.01 ± 0.13	1.98 ± 0.14	2.00 ± 0.15
		Post	1.99 ± 0.17	1.95 ± 0.15	1.98 ± 0.14
	Pre-PHV	Pre	1.98 ± 0.04	2.07 ± 0.09	2.07 ± 0.14
		Post	2.02 ± 0.06	2.11 ± 0.09	2.05 ± 0.12
	Circa-PHV	Pre	2.05 ± 0.16	2.04 ± 0.20	2.03 ± 0.09
		Post	2.02 ± 0.14	2.00 ± 0.11	1.98 ± 0.19
	Post-PHV	Pre	1.95 ± 0.07	1.93 ± 0.10	1.96 ± 0.18
		Post	1.93 ± 0.10	1.89 ± 0.14	1.97 ± 0.17
20 m (s)	All	Pre	3.52 ± 0.26	3.46 ± 0.28	3.49 ± 0.30
		Post	3.45 ± 0.23 *	3.40 ± 0.23 *	3.46 ± 0.27
	Pre-PHV	Pre	3.47 ± 0.04	3.70 ± 0.22	3.66 ± 0.29
		Post	3.50 ± 0.09	3.70 ± 0.15	3.56 ± 0.20
	Circa-PHV	Pre	3.60 ± 0.30	3.57 ± 0.37	3.55 ± 0.19
		Post	3.52 ± 0.26 *	3.45 ± 0.22	3.49 ± 0.16 *
	Post-PHV	Pre	3.40 ± 0.15	3.37 ± 0.20	3.42 ± 0.35
		Post	3.33 ± 0.18 *	3.31 ± 0.19 *	3.41 ± 0.34

Note: * = significantly different to pre-test (*p* < 0.05).

**Table 4:** Pre- and post-jump mean ± SD for training and maturation groups.

Metric	Maturation Group	Test	Control Mean ± SD	Traditional Mean ± SD	Progressive Mean ± SD
HJD (m)	All	Pre	1.55 ± 0.21	1.65 ± 0.18	1.59 ± 0.25
		Post	1.65 ± 0.22 *	1.74 ± 0.18 *	1.70 ± 0.23 *
	Pre-PHV	Pre	1.55 ± 0.12	1.50 ± 0.15	1.46 ± 0.17
		Post	1.63 ± 0.16	1.61 ± 0.08	1.61 ± 0.08
	Circa-PHV	Pre	1.54 ± 0.24	1.57 ± 0.20	1.60 ± 0.23
		Post	1.62 ± 0.26	1.72 ± 0.20 *	1.68 ± 0.14
	Post-PHV	Pre	1.59 ± 0.19	1.71 ± 0.14	1.63 ± 0.28
		Post	1.73 ± 0.14 *	1.78 ± 0.17 *	1.73 ± 0.30
HJND (m)	All	Pre	1.48 ± 0.21	1.58 ± 0.17	1.52 ± 0.25
		Post	1.56 ± 0.22 *	1.66 ± 0.18 *	1.63 ± 0.24 *
	Pre-PHV	Pre	1.45 ± 0.14	1.48 ± 0.15	1.41 ± 0.12
		Post	1.51 ± 0.11	1.54 ± 0.09	1.56 ± 0.08 *
	Circa-PHV	Pre	1.46 ± 0.24	1.48 ± 0.19	1.50 ± 0.22
		Post	1.52 ± 0.26	1.62 ± 0.20	1.60 ± 0.16
	Post-PHV	Pre	1.53 ± 0.20	1.64 ± 0.14	1.56 ± 0.29
		Post	1.65 ± 0.14 *	1.71 ± 0.18 *	1.66 ± 0.31
TJ Score	All	Pre	13.9 ± 2.6	11.6 ± 3.0	12.0 ± 2.9
		Post	13.1 ± 2.8	12.4 ± 3.0	13.5 ± 2.7 *
	Pre-PHV	Pre	15.0 ± 3.0	13.0 ± 0.8	11.8 ± 1.5
		Post	12.7 ± 2.5	12.0 ± 3.4	14.8 ± 1.5
	Circa-PHV	Pre	13.8 ± 2.1	12.4 ± 3.0	11.5 ± 3.2
		Post	12.6 ± 2.4	14.1 ± 3.1	12.7 ± 3.6
	Post-PHV	Pre	13.8 ± 3.5	11.0 ± 3.2	12.1 ± 2.9
		Post	14.1 ± 3.6	11.8 ± 2.8	13.5 ± 2.1

Note: * = significantly different to pre-test (*p* < 0.05).

**Table 5:** Percentage change (90% confidence limit (CL)) in sprint metrics within maturational groups across control, traditional and progressive training groups.

Metric	Maturation	Control		Traditional		Progressive	
		%diff ± CL	(ES ± CL)	%diff ± CL	(ES ± CL)	%diff ± CL	(ES ± CL)
5 m (s)	All	0.0 ± 1.5	(−0.01 ± 0.22)	0.1 ± 1.4	(0.02 ± 0.21)	0.1 ± 1.1	(0.01 ± 0.16)
	Pre-PHV	3.3 ± 10.4	(0.57 ± 1.72)	4.4 ± 5.2	(0.70 ± 0.83)	−2.1 ± 2.9	(−0.27 ± 0.37)
	Circa-PHV	−0.3 ± 2.0	(−0.03 ± 0.25)	−1.1 ± 3.3	(−0.11 ± 0.35)	−0.1 ± 1.9	(−0.03 ± 0.39)
	Post-PHV	−0.9 ± 2.4	(−0.24 ± 0.62)	−0.4 ± 1.6	(−0.07 ± 0.30)	0.7 ± 1.7	(0.09 ± 0.20)
10 m (s)	All	−0.7 ± 1.1	(−0.10 ± 0.17)	−1.4 ± 1.8	(−0.20 ± 0.26)	−0.7 ± 1.6	(−0.10 ± 0.21)
	Pre-PHV	2.0 ± 6.6	(0.60 ± 1.91)	2.0 ± 3.2	(0.32 ± 0.51)	−1.1 ± 2.7	(−0.12 ± 0.30)
	Circa-PHV	−1.1 ± 1.6	(−0.13 ± 0.20)	−1.5 ± 3.3	(−0.14 ± 0.30)	−2.6 ± 4.4	(−0.55 ± 0.89)
	Post-PHV	−0.9 ± 1.7	(−0.24 ± 0.42)	−2.1 ± 2.7	(−0.41 ± 0.51)	0.6 ± 1.6	(0.06 ± 0.18)
20 m (s)	All	−1.8 ± 1.1	(−0.25 ± 0.15) *	−1.8 ± 1.1	(−0.23 ± 0.13) *	−1.1 ± 1.1	(−0.12 ± 0.13)
	Pre-PHV	0.8 ± 5.0	(0.40 ± 2.33)	0.0 ± 3.4	(0.00 ± 0.41)	−2.7 ± 3.2	(−0.25 ± 0.29)
	Circa-PHV	−2.2 ± 1.7	(−0.26 ± 0.19) *	−3.1 ± 3.3	(−0.27 ± 0.28)	−1.6 ± 1.2	(−0.27 ± 1.21) *
	Post-PHV	−2.1 ± 1.5	(−0.43 ± 0.30) *	−1.7 ± 1.2	(−0.28 ± 0.20) *	−0.3 ± 1.9	(−0.03 ± 0.19)

Note: %diff = percentage difference in means; CL = 90% confidence limits; ES = effect size; * = significant difference in pre/post means (*p* < 0.05).

**Table 6:** Percentage change (90% CL) in jump metrics within maturational groups across control, traditional and progressive training groups.

Metric	Maturation	Control		Traditional		Progressive	
		%diff ± CL	(ES ± CL)	%diff ± CL	(ES ± CL)	%diff ± CL	(ES ± CL)
HJD	All	6.4 ± 3.0	(0.43 ± 0.20) *	6.0 ± 2.1	(0.52 ± 0.19) *	6.7 ± 2.0	(0.41 ± 0.13) *
	Pre-PHV	4.8 ± 5.9	(0.34 ± 0.42)	7.6 ± 7.5	(0.50 ± 0.50)	10.8 ± 10.7	(0.63 ± 0.62)
	Circa-PHV	5.1 ± 4.5	(0.29 ± 0.26)	10.1 ± 4.9	(0.64 ± 0.32) *	5.4 ± 4.6	(0.36 ± 0.30)
	Post-PHV	9.3 ± 5.8	(0.63 ± 0.40) *	4.0 ± 2.7	(0.45 ± 0.30) *	6.5 ± 2.2	(0.35 ± 0.12) *
HJND	All	5.6 ± 2.9	(0.35 ± 0.18) *	5.4 ± 2.6	(0.45 ± 0.22) *	7.2 ± 2.1	(0.41 ± 0.12) *
	Pre-PHV	4.3 ± 11.3	(0.25 ± 0.63)	4.3 ± 7.9	(0.29 ± 0.53)	11.0 ± 6.2	(0.85 ± 0.49) *
	Circa-PHV	4.2 ± 3.8	(0.22 ± 0.20)	9.9 ± 8.2	(0.60 ± 0.50)	7.3 ± 5.9	(0.45 ± 0.37)
	Post-PHV	8.5 ± 6.5	(0.55 ± 0.43) *	3.8 ± 2.9	(0.42 ± 0.32) *	6.2 ± 2.1	(0.30 ± 0.10) *
TJ Score	All	−6.4 ± 12.3	(−0.35 ± 0.61)	6.8 ± 9.9	(0.23 ± 0.33)	13.1 ± 8.0	(0.47 ± 0.29) *
	Pre-PHV	−15.6 ± 84.9	(−0.48 ± 1.73)	−11.0 ± 49.7	(−1.34 ± 4.66)	25.8 ± 22.0	(1.29 ± 1.11)
	Circa-PHV	−8.9 ± 13.4	(−0.60 ± 0.81)	14.2 ± 29.1	(0.46 ± 0.89)	10.2 ± 20.8	(0.30 ± 0.59)
	Post-PHV	1.9 ± 36.1	(0.07 ± 1.09)	8.4 ± 11.7	(0.25 ± 0.35)	12.9 ± 10.4	(0.45 ± 0.37) *

Note: %diff = percentage difference in means; CL = 90% confidence limits; ES = effect size; * = significant difference in pre/post means (*p* < 0.05); HJD = Horizontal jump dominant leg; HJND = Horizontal jump non-dominant leg.

**Table 7:** Percentage difference (90% CL) in sprint change scores within maturation groups and between training groups.

Metric	Maturation	Control vs. Traditional		Control vs. Progressive		Traditional vs. Progressive	
		%diff ± CL	(ES ± CL)	%diff ± CL	(ES ± CL)	%diff ± CL	(ES ± CL)
5 m (s)	All	0.4 ± 2.1	(0.07 ± 0.35)	0.2 ± 1.9	(0.03 ± 0.27)	0.0 ± 1.9	(0.00 ± 0.27)
	Pre-PHV	1.0 ± 9.9	(0.23 ± 2.14)	−5.3 ± 11.1	(−0.90 ± 1.75)	−6.2 ± 5.4	(−1.14 ± 0.94)
	Circa-PHV	−0.8 ± 3.7	(−0.10 ± 0.46)	0.1 ± 2.7	(0.02 ± 0.40)	−0.8 ± 3.7	(−0.10 ± 0.56)
	Post-PHV	0.5 ± 2.7	(0.12 ± 0.59)	1.6 ± 2.8	(0.23 ± 0.39)	1.1 ± 2.3	(0.16 ± 0.33)
10 m (s)	All	−0.9 ± 2.5	(−0.14 ± 0.45)	−0.2 ± 2.4	(−0.03 ± 0.33)	0.5 ± 2.8	(0.07 ± 0.37)
	Pre-PHV	0.0 ± 6.2	(0.00 ± 1.30)	−3.1 ± 6.0	(−0.51 ± 0.95)	−3.1 ± 3.6	(−0.53 ± 0.60)
	Circa-PHV	−0.4 ± 3.5	(−0.05 ± 0.42)	−1.6 ± 4.7	(−0.24 ± 0.69)	−0.4 ± 3.5	(−0.05 ± 0.74)
	Post-PHV	−1.2 ± 3.0	(−0.26 ± 0.65)	1.5 ± 2.2	(0.20 ± 0.29)	2.7 ± 3.0	(0.38 ± 0.43)
20 m (s)	All	0.4 ± 1.5	(0.06 ± 0.21)	1.0 ± 1.8	(0.12 ± 0.21)	0.9 ± 1.8	(0.11 ± 0.21)
	Pre-PHV	−0.9 ± 4.8	(−0.14 ± 0.75)	−3.5 ± 4.7	(−0.49 ± 0.63)	−2.9 ± 4.2	(−0.11 ± 0.15)
	Circa-PHV	−0.9 ± 3.6	(−0.10 ± 0.39)	0.7 ± 2.0	(0.09 ± 0.28)	−0.9 ± 3.6	(−0.10 ± 0.45)
	Post-PHV	0.4 ± 1.8	(0.07 ± 0.33)	1.7 ± 2.3	(0.21 ± 0.27)	1.3 ± 2.2	(0.17 ± 0.27)

Note: %diff = percentage difference in means; CL = 90% confidence limits; ES = effect size.

**Table 8:** Percentage difference (90%CL) in jump change scores within maturation groups and between training groups.

Metric	Maturation	Control vs. Traditional		Control vs. Progressive		Traditional vs. Progressive	
		%diff ± CL	(ES ± CL)	%diff ± CL	(ES ± CL)	%diff ± CL	(ES ± CL)
HJD	All	−1.4 ± 3.8	(−0.11 ± 0.29)	0.8 ± 3.6	(0.05 ± 0.23)	1.1 ± 2.9	(0.08 ± 0.21)
	Pre-PHV	2.6 ± 8.1	(0.25 ± 0.76)	5.7 ± 10.6	(0.47 ± 0.86)	3.0 ± 11.2	(0.25 ± 0.90)
	Circa-PHV	4.8 ± 6.3	(0.31 ± 0.40)	0.4 ± 6.2	(0.02 ± 0.39)	−4.2 ± 6.3	(−0.32 ± 0.45)
	Post-PHV	−4.8 ± 6.3	(−0.46 ± 0.57)	−2.5 ± 6.1	(−0.16 ± 0.37)	2.4 ± 3.4	(0.17 ± 0.27)
HJND	All	−1.6 ± 3.8	(−0.11 ± 0.27)	1.6 ± 3.3	(0.09 ± 0.20)	1.8 ± 3.1	(0.12 ± 0.21)
	Pre-PHV	0.0 ± 11.0	(0.00 ± 0.97)	6.4 ± 11.1	(0.63 ± 1.07)	6.4 ± 8.7	(0.59 ± 0.79)
	Circa-PHV	5.4 ± 8.8	(0.32 ± 0.51)	2.9 ± 6.8	(0.18 ± 0.40)	−2.3 ± 9.5	(−0.17 ± 0.64)
	Post-PHV	−4.3 ± 7.0	(−0.40 ± 0.62)	−2.1 ± 6.8	(−0.12 ± 0.37)	2.3 ± 3.5	(0.15 ± 0.23)
TJ Score	All	9.6 ± 16.6	(1.35 ± 0.58)	22.8 ± 15.1	(0.86 ± 0.59) *	7.7 ± 13.2	(0.27 ± 0.45)
	Pre-PHV	5.4 ± 78.4	(0.32 ± 3.50)	49.0 ± 94.0	(1.77 ± 2.95)	41.3 ± 50.3	(2.79 ± 3.29)
	Circa-PHV	25.4 ± 31.5	(1.14 ± 1.39)	20.9 ± 24.4	(0.77 ± 0.88)	−3.5 ± 34.7	(−0.13 ± 1.04)
	Post-PHV	6.3 ± 37.7	(0.19 ± 1.02)	9.9 ± 37.5	(0.35 ± 1.19)	3.3 ± 15.0	(0.11 ± 0.47)

Note: * = significant difference between training groups (*p* < 0.05); %diff = percentage difference in means; CL = 90% confidence limits; ES = effect size; HJD = horizontal jump dominant leg; HJND = horizontal jump non-dominant leg.

## Appendix A.

**Table A1:** Modified TJ Rubric Derived from Fort-Vanmeerhaeghe et al. (2017).

Phase of Jump	Criterion	View	None (0)	Small (1)	Large (2)
Knee and thigh motion	1. Lower Extremity valgus at landing	F	No valgus	Slight Valgus	Obvious valgus: Both knees touch
	2. Thighs do not reach parallel (peak of jump)	L	The knees are higher or at the same level as the hips	The middle of the knees are at a lower level than the middle of the hips	The whole knees are under the entire hips
	3. Thighs not equal side to-side during flight	F	Thighs equal side to side	Thighs slightly unequal side to side	Thighs completely unequal side to side (one knee over the other)
Foot position during landing	4. Foot placement not shoulder width apart	F	Foot placement exactly shoulder width apart	Foot placement less than shoulder width but more than one foot width of one another	Foot placement less than one foot width of one another
	5. Foot placement not parallel (front to back)	L	Foot placement parallel (end of feet within big toe length)	Foot placement unparalleled (end of feet greater than big toe length, but less than half their foot)	Foot placement obviously unparalleled (end of feet greater than half their foot length)
	6. Foot contact timing not equal (Asymmetrical landing)	F	Foot contact timing equal side-to-side	Foot contact timing slightly unequal	Foot contact timing completely unequal
	7. Excessive landing contact noise	F/L	Subtle noise at landing (landing on balls of feet)	Audible noise at landing (heels touch ground during landing but controlled)	Loud and pronounced noise at landing (entire foot and heel touch ground during landing with lack of control)
Plyometric ability	8. Pause between jumps	F/L	Reactive and reflex jumps	Small pause between jumps	Large pause between jumps or double contact between jumps
	9. Technique declines prior ten seconds	F/L	No decline in technique	Decline in technique after five secs	Decline in technique before five seconds
	10. Does not land in same foot print (Consistent point of landing)	F/L	Touches tape with both feet	One foot on tape, one foot not touching tape	Both feet miss tape

Note: F = Frontal view; L = Lateral view.

## Appendix B.

**Table A2:** Pre and Post Mean ± SD Kinematic Measures for Training and Maturation Groups.

Variable	Group	Control		Traditional		Progressive	
		Pre ± SD	Post ± SD	Pre ± SD	Post ± SD	Pre ± SD	Post ± SD
SL S1 (m)	All	1.04 ± 0.09	1.08 ± 0.14	1.08 ± 0.11	1.12 ± 0.14 ^‡^	1.06 ± 0.11	1.07 ± 0.11
	Pre	1.00 ± 0.05	1.03 ± 0.08	-	-	0.98 ± 0.06	1.02 ± 0.07
	Circa	1.04 ± 0.10	1.04 ± 0.17	1.11 ± 0.05	1.20 ± 0.20	0.96 ± 0.11	0.99 ± 0.12
	Post	1.07 ± 0.07	1.15 ± 0.08	1.09 ± 0.10	1.12 ± 0.08 *	1.11 ± 0.10	1.11 ± 0.09
SL S2 (m)	All	1.15 ± 0.19	1.20 ± 0.11 *	1.22 ± 0.11	1.21 ± 0.13	1.17 ± 0.12	1.20 ± 0.13
	Pre	1.14 ± 0.09	1.14 ± 0.07	-	-	1.07 ± 0.08	1.12 ± 0.15
	Circa	1.14 ± 0.12	1.19 ± 0.10 *	1.21 ± 0.12	1.17 ± 0.10 ^†^	1.13 ± 0.08	1.17 ± 0.11
	Post	1.17 ± 0.11	1.25 ± 0.13 *	1.24 ± 0.10	1.24 ± 0.12 ^†^	1.22 ± 0.13	1.23 ± 0.12 ^†^
SL S3 (m)	All	1.27 ± 0.09	1.23 ± 0.10	1.32 ± 0.14	1.31 ± 0.11	1.29 ± 0.13	1.32 ± 0.17
	Pre	1.27 ± 0.05	1.24 ± 0.05	-	-	1.22 ± 0.11	1.29 ± 0.09
	Circa	1.26 ± 0.10	1.27 ± 0.09	1.34 ± 0.11	1.29 ± 0.11	1.25 ± 0.09	1.30 ± 0.16
	Post	1.30 ± 0.09	1.37 ± 0.10 *	1.34 ± 0.13	1.34 ± 0.10 ^†^	1.33 ± 0.13	1.33 ± 0.11 ^†^
SL S4 (m)	All	1.35 ± 0.10	1.35 ± 0.08	1.36 ± 0.11	1.34 ± 0.14	1.38 ± 0.16	1.39 ± 0.17
	Pre	1.30 ± 0.10	1.27 ± 0.06	-	-	1.25 ± 0.10	1.35 ± 0.09
	Circa	1.32 ± 0.09	1.34 ± 0.08	-	-	1.30 ± 0.05	1.31 ± 0.07
	Post	1.43 ± 0.07	1.42 ± 0.05	1.39 ± 0.09	1.37 ± 0.12	1.44 ± 0.17	1.43 ± 0.14
SL 15m (m)	All	1.71 ± 0.10	1.73 ± 0.11	1.76 ± 0.12	1.76 ± 0.12	1.70 ± 0.14	1.75 ± 0.12 *
	Pre	1.68 ± 0.09	1.69 ± 0.12	-	-	1.67 ± 0.05	1.67 ± 0.06
	Circa	1.67 ± 0.08	1.70 ± 0.08	1.75 ± 0.08	1.76 ± 0.12	1.68 ± 0.03	1.77 ± 0.03 *
	Post	1.78 ± 0.10	1.78 ± 0.14	1.78 ± 0.12	1.78 ± 0.10	1.72 ± 0.18	1.76 ± 0.14
CT S1 (s)	All	0.22 ± 0.02	0.22 ± 0.03 *	0.25±0.19	0.22 ± 0.03	0.22 ± 0.03	0.22 ± 0.02
	Pre	0.21 ± 0.04	0.21 ± 0.04	0.22 ± 0.01	0.22 ± 0.03	0.22 ± 0.04	0.22 ± 0.03
	Circa	0.22 ± 0.03	0.22 ± 0.03	0.21 ± 0.03	0.22 ± 0.03	0.21 ± 0.02	0.21 ± 0.02
	Post	0.22 ± 0.01	0.23 ± 0.02 *	0.28 ± 0.24	0.22 ± 0.03	0.23 ± 0.03	0.23 ± 0.02 ^†^
CT S2 (s)	All	0.22 ± 0.10	0.20 ± 0.02	0.20 ± 0.02	0.20 ± 0.02	0.20 ± 0.02	0.20 ± 0.02
	Pre	0.20 ± 0.02	0.21 ± 0.03	0.20 ± 0.02	0.20 ± 0.01	0.20 ± 0.02	0.19 ± 0.02
	Circa	0.24 ± 0.13	0.20 ± 0.02	0.20 ± 0.03	0.19 ± 0.01 *^‡^	0.19 ± 0.02	0.19 ± 0.02
	Post	0.20 ± 0.02	0.19 ± 0.01	0.19 ± 0.01	0.20 ± 0.02	0.20 ± 0.02	0.21 ± 0.02
CT S3 (s)	All	0.19 ± 0.02	0.19 ± 0.02	0.18 ± 0.02	0.18 ± 0.02	0.19 ± 0.02	0.18 ± 0.02
	Pre	0.18 ± 0.02	0.18 ± 0.03	0.19 ± 0.02	0.19 ± 0.01	0.19 ± 0.03	0.18 ± 0.02
	Circa	0.19 ± 0.02	0.19 ± 0.02	0.18 ± 0.02	0.18 ± 0.01	0.18 ± 0.02	0.18 ± 0.01
	Post	0.19 ± 0.01	0.19 ± 0.01	0.18 ± 0.01	0.18 ± 0.02	0.19 ± 0.02	0.19 ± 0.02
CT S4 (s)	All	0.17 ± 0.02	0.17 ± 0.02	0.17 ± 0.02	0.17 ± 0.01	0.18 ± 0.02	0.17 ± 0.02
	Pre	0.16 ± 0.03	0.17 ± 0.02	0.17 ± 0.02	0.17 ± 0.01	0.18 ± 0.03	0.16 ± 0.01
	Circa	0.18 ± 0.02	0.17 ± 0.02	0.18 ± 0.02	0.16 ± 0.02 *	0.17 ± 0.02	0.17 ± 0.02
	Post	0.17 ± 0.02	0.17 ± 0.01	0.17 ± 0.02	0.17 ± 0.01	0.18 ± 0.02	0.18 ± 0.02
CT 15m (s)	All	0.18 ± 0.10	0.15 ± 0.02	0.16 ± 0.02	0.15 ± 0.01 *^‡^	0.16 ± 0.02	0.15 ± 0.02
	Pre	0.15 ± 0.02	0.15 ± 0.02	0.17 ± 0.02	0.15 ± 0.01	0.16 ± 0.03	0.15 ± 0.02
	Circa	0.20 ± 0.13	0.15 ± 0.02	0.16 ± 0.02	0.14 ± 0.02 *^‡^	0.15 ± 0.02	0.15 ± 0.01
	Post	0.15 ± 0.01	0.15 ± 0.02	0.15 ± 0.02	0.15 ± 0.01	0.16 ± 0.02	0.16 ± 0.02
FT S1 (s)	All	0.05 ± 0.02	0.05 ± 0.01	0.05 ± 0.02	0.05 ± 0.02	0.05 ± 0.01	0.05 ± 0.01
	Pre	0.04 ± 0.02	0.05 ± 0.02	0.04 ± 0.01	0.04 ± 0.02	0.05 ± 0.01	0.05 ± 0.01
	Circa	0.05 ± 0.01	0.05 ± 0.01	0.05 ± 0.02	0.06 ± 0.02 *^†^	0.05 ± 0.01	0.05 ± 0.02
	Post	0.05 ± 0.02	0.05 ± 0.01	0.05 ± 0.02	0.05 ± 0.01	0.04 ± 0.02	0.04 ± 0.01
FT S2 (s)	All	0.06 ± 0.02	0.06 ± 0.01	0.06 ± 0.02	0.06 ± 0.02	0.06 ± 0.01	0.06 ± 0.02
	Pre	0.06 ± 0.00	0.05 ± 0.01	0.05 ± 0.01	0.05 ± 0.02	0.05 ± 0.00	0.06 ± 0.01
	Circa	0.06 ± 0.02	0.06 ± 0.02	0.06 ± 0.01	0.06 ± 0.02	0.05 ± 0.01	0.05 ± 0.02
	Post	0.06 ± 0.01	0.06 ± 0.01	0.06 ± 0.02	0.06 ± 0.02	0.06 ± 0.02	0.06 ± 0.02
FT S3 (s)	All	0.07 ± 0.01	0.07 ± 0.01	0.07 ± 0.02	0.08 ± 0.08	0.07 ± 0.01	0.07 ± 0.01
	Pre	0.07 ± 0.01	0.07 ± 0.01	0.07 ± 0.01	0.15 ± 0.22	0.07 ± 0.01	0.08 ± 0.02
	Circa	0.07 ± 0.01	0.07 ± 0.01	0.07 ± 0.01	0.07 ± 0.02	0.07 ± 0.01	0.07 ± 0.01
	Post	0.07 ± 0.02	0.07 ± 0.01 *	0.07 ± 0.02	0.07 ± 0.01	0.07 ± 0.02	0.07 ± 0.01
FT 15m (s)	All	0.09 ± 0.01	0.10 ± 0.01	0.09 ± 0.02	0.10 ± 0.02 *	0.09 ± 0.02	0.10 ± 0.01 *
	Pre	0.10 ± 0.02	0.11 ± 0.01	0.09 ± 0.01	0.10 ± 0.02	0.10 ± 0.02	0.10 ± 0.01
	Circa	0.09 ± 0.01	0.09 ± 0.01	0.09 ± 0.02	0.10 ± 0.01 *	0.09 ± 0.02	0.10 ± 0.01 *^†^
	Post	0.09 ± 0.01	0.10 ± 0.02	0.10 ± 0.01	0.10 ± 0.02	0.09 ± 0.02	0.10 ± 0.01
SF S1 (Hz)	All	3.82 ± 0.40	3.73 ± 0.37	3.74 ± 0.51	3.82 ± 0.44 ^†^	3.86 ± 0.45	3.80 ± 0.32
	Pre	3.94 ± 0.47	3.91 ± 0.35	3.92 ± 0.36	3.90 ± 0.71	3.84 ± 0.56	3.73 ± 0.32
	Circa	3.81 ± 0.44	3.77 ± 0.35	3.90 ± 0.45	3.70 ± 0.40	4.03 ± 0.40	3.92 ± 0.38
	Post	3.80 ± 0.34	3.57 ± 0.38	3.63 ± 0.55	3.85 ± 0.40 ^†^	3.77 ± 0.45	3.75 ± 0.28
SF S2 (Hz)	All	3.80 ± 0.48	3.92 ± 0.34	3.90 ± 0.31	3.88 ± 0.46	3.96 ± 0.34	3.90 ± 0.33
	Pre	3.91 ± 0.34	3.91 ± 0.29	4.00 ± 0.17	3.60 ± 0.93	3.99 ± 0.36	4.06 ± 0.35
	Circa	3.68 ± 0.57	3.88 ± 0.37	3.80 ± 0.34	4.01 ± 0.36 ^‡^	4.18 ± 0.35	4.02 ± 0.33 ^†^
	Post	3.98 ± 0.25	4.00 ± 0.35	3.91 ± 0.32	3.89 ± 0.34	3.84 ± 0.28	3.80 ± 0.32
SF S3 (Hz)	All	3.91 ± 0.26	3.98 ± 0.40	3.96 ± 0.35	3.98 ± 0.32	3.99 ± 0.33	3.99 ± 0.37
	Pre	4.10 ± 0.08	4.36 ± 0.85	3.96 ± 0.55	3.91 ± 0.45	3.92 ± 0.41	3.89 ± 0.58
	Circa	3.89 ± 0.25	3.93 ± 0.34	3.94 ± 0.35	3.92 ± 0.27	4.10 ± 0.31	4.10 ± 0.38
	Post	3.89 ± 0.31	3.91 ± 0.22	3.97 ± 0.33	4.01 ± 0.32	3.96 ± 0.33	3.97 ± 0.32
Sf 15m (Hz)	All	3.93 ± 0.43	4.05 ± 0.33	4.05 ± 0.36	4.10 ± 0.38	4.10 ± 0.40	4.24 ± 0.90
	Pre	3.96 ± 0.23	4.01 ± 0.30	3.85 ± 0.32	4.01 ± 0.32	3.90 ± 0.44	4.06 ± 0.16
	Circa	3.84 ± 0.52	4.06 ± 0.31	4.13 ± 0.43	4.13 ± 0.38	4.18 ± 0.48	4.01 ± 0.30 ^†^
	Post	4.08 ± 0.26	4.04 ± 0.43	4.06 ± 0.34	4.11 ± 0.42	4.10 ± 0.33	4.43 ± 1.19

Note: * = significant difference (*p* < 0.05) pre vs. post; † = significant difference (*p* < 0.05) to control change scores, ‡ = significant difference (*p* < 0.05) to traditional change scores.
